# Advanced extraction techniques for sustainable recovery of health-promoting compounds from carob leaves

**DOI:** 10.1016/j.ultsonch.2025.107710

**Published:** 2025-12-06

**Authors:** Simona Serio, Valentina Santoro, Anna Lisa Piccinelli, Rita Celano, Luca Rastrelli

**Affiliations:** a Department of Pharmacy, University of Salerno 84084 Fisciano, Salerno, Italy; b PhD Program in Drug Discovery and Development, University of Salerno 84084 Fisciano, Salerno, Italy; c National Biodiversity Future Center (NBFC), 90133 Palermo, PA, Italy

**Keywords:** Agricultural co-products, Green extraction, Responsible consumption and production − SDG12, Good health and well-being − SDG3, Design of experiments, Functional ingredients

## Abstract

Carob (*Ceratonia siliqua*) leaves (CSL) are an underutilized source of health-promoting compounds, including siliquapyranone, 1,2,3,6-tetragalloylglucose and myricitrin. CSL and its main compounds revealed remarkable antioxidant and hypoglycaemic potential. This study presents a systematic optimization of pressurized hot water extraction (PHWE) and probe-based ultrasound-assisted extraction (probe-UAE) as sustainable strategies for the recovery of CSL bioactive compounds. Using response surface methodology combined with UHPLC-UV profiling, the main parameters influencing extraction efficiency and compound stability were identified. Optimal PHWE (98 °C, three 5 min-cycles) and probe-UAE (ethanol 52 %, 100 g L^–1^, 20 min) conditions ensured exhaustive recovery of target compounds with negligible formation of extraction artefacts (gallic acid and digalloylglucoses), preserving antioxidant and α-glucosidase inhibitory activities. Compared with conventional techniques, PHWE and probe-UAE demonstrated a low environmental footprint, reducing extraction time, solvent use and environmental impact, while improving extraction yield and extract quality. PHWE generated fully aqueous extracts suitable for direct application, whereas probe-UAE offered high energy efficiency and operational simplicity. These findings demonstrate the potential of green extraction techniques for the sustainable valorization of carob leaves, providing high-value bioactive extracts and supporting the development of healthy food products. This study offers novel insights into the exploitation of untapped plant biomasses and provides a practical approach for integrating green innovations into the food and nutraceutical sectors.

## 1 Introduction

The growing interest in plant-based products is driven by their health benefits, mainly attributed to bioactive and functional compounds. Increasing evidence highlights the role of plant-derived bioactive compounds in promoting health and managing noncommunicable diseases, such as dyslipidemia, type-2 diabetes, stroke, cancer, cardiovascular and neurodegenerative diseases. Their recovery from plant materials has therefore gained significant industrial relevance, particularly for use in novel functional foods, dietary supplements, cosmetics, and agricultural products [[1], [2], [3]]. The sustainable recovery of valuable bioactive compounds from plant biomass is gaining prominence within the framework of the United Nations Sustainable Development Goals (SDGs), particularly SDG 12 (responsible consumption and production), SDG 13 (climate action) and SDG 3 (good health and well-being). Although conventional extraction techniques (maceration, hydro-distillation, Soxhlet extraction) are still widely applied, they are time- and energy-consuming, require large amounts of harmful organic solvents, and often lead to insufficient recovery while generating significant quantity of waste [4]. These drawbacks disagree with the six principles of green extraction, which aim to develop efficient, safe and sustainable processes that minimize processing time, solvent use, energy demand, waste generation and environmental impact, while enhancing extraction efficiency and extract quality [[5], [6], [7]]. In this context, advanced extraction techniques, such as pressurized hot water extraction (PHWE) and ultrasound-assisted extraction (UAE), have emerged as viable alternatives to conventional methods and are regarded as sustainable processing approaches aligned with green extraction principles [4,6,8,9]. (PHWE), also called subcritical water extraction (SWE), allows rapid, automated, solvent-free extraction and efficient recovery of polar and semi-polar compounds while yielding high-quality extracts suitable for direct application [10,11]. It has been established as a specialized technique for extracting polyphenols, proteins and polysaccharides from different natural sources [12,13]. However, PHWE requires specific operating conditions that may induce undesirable reactions or degradation of thermolabile compounds [[13], [14], [15]]. Also UAE is widely applied for polyphenol recovery [14,16,17]. It is regarded as one of the simplest and most cost-effective extraction approaches, capable of enhancing extraction yield while significantly reducing organic solvent use and extraction time. Moreover, UAE operates at lower temperatures, preserving thermolabile compounds [16,18]. Nonetheless, UAE still involves organic solvents, although GRAS (generally recognized as safe) for most applications, and post-extraction steps, such as filtration and solvent evaporation/recycling steps [14].

Among promising plant sources, the leaves of *Ceratonia siliqua* (CSL). a resilient Mediterranean species recently revaluated for its ecological and nutritional significance [19,20], represent an untapped biomass rich in health-promoting phytochemicals. A recent study [21] reported high levels of *n*-galloylated glucoses and flavonol-glycosides, with siliquapyranone (Sil), 1,2,3,6-tetragalloylglucose (TeGG) and myricitrin (Myr) identified as key CSL markers. CSL extracts and its main compounds revealed remarkable antioxidant and hypoglycaemic properties. In particular, CSL extracts and TeGG exhibited potent and selective inhibition of α-glucosidase (α-GLU) [21], a crucial enzymatic target in the management of postprandial hyperglycemia. These findings highlight CSL as a valuable source of bioactive compounds for the formulation of novel foods, functional products, and dietary supplements for the prevention and adjunctive treatment of type 2 diabetes mellitus. To fully valorize carob leaves as a high-value co-product and contribute to the economic value of carob-chain, efficient and sustainable extraction processes are essential. Despite the strong health-promoting potential of carob leaves as natural source of bioactive compounds, no study to date has systematically optimized the extraction parameters using advanced green technologies. The high diversity and complexity of plant biomass require an accurate selection of operating parameters, as they significantly affect the process effectiveness. A fine-tuning of extraction conditions is crucial not only to maximize target compound recovery and reduce environmental impact [14,22], but also to ensure extract quality in terms of naturalness, absence of artifacts or degradation products, and high functionality of the extracts [8,14].

This study focuses on the design and optimization of PHWE and UAE using a probe system (probe-UAE) for the sustainable recovery of bioactive compounds from CSL. PHWE and probe-UAE were investigated and optimized through response surface methodology (RSM) and quali-quantitative UHPLC-UV profiling of CSL extracts, aiming to maximize the recovery and extract content of CSL markers, Sil, TeGG and Myr, while simultaneously minimizing their degradation during PHWE and UAE processes. Furthermore, the biological functionality of the optimized extracts was validated by assessing their antioxidant capacity (ABTS assay), hypoglycaemic potential (α-GLU inhibitory assays), and *in vitro* cytotoxicity. Finally, the performance of these extraction techniques was compared with conventional methods, specifically Soxhlet extraction and maceration. Overall, this work provides the first systematic optimization of PHWE and UAE for CSL, contributing to the sustainable valorization of carob leaves within a circular bioeconomy framework.

## 2 Materials and methods

### 2.1 Materials and reagents

MS-grade acetonitrile (MeCN), methanol, and water were supplied by Romil (Deltek, Italy). Ultrapure water (18 MΩ) was prepared using a Milli-Q purification system (Millipore, Bedford, USA). Analytical-grade ethanol, MS-grade formic acid, 2,2’-azinobis(3-ethylbenzothiazoline-6-sulphonic acid) diammonium salt (ABTS), 4-nitrophenyl-β-D-glucopyranoside (pNPG), 5-hydroxymethyl-2-furaldehyde, 6-hydroxy-2,5,7,8-tetramethylchromane-2-carboxylic acid (Trolox), disodium hydrogen phosphate (Na_2_HPO_4_), Folin & Ciocalteu’s phenol reagent (FC), potassium dihydrogen phosphate (KH_2_PO_4_), potassium persulfate (K_2_S_2_O_8_), pyrogallol, sodium carbonate (Na_2_CO_3_), were purchased from Merck Chemicals (Milan, Italy). The enzyme α-glucosidase from *Saccharomyces cerevisiae* (EC 3.2.1.20, Type I, lyophilized powder, ≥ 10 units/mg protein) was obtained from Merck Chemicals. Reference standards of myricitrin (purity ≥ 98 %) (Myr), gallic acid (purity ≥ 98 %), acarbose (purity ≥ 98 %) and quercetin (purity ≥ 95 %) were acquired from Merck Chemicals. 1,2,3,6-Tetragalloylglusose (TeGG) and siliquapyranone (Sil) were previously isolated by *C. siliqua* leaves [21].

### 2.2 Samples

Samples of *C. siliqua* leaves (CSL) from the Amele variety were monthly harvested during summer 2023 (June-August) from carob trees at Masseria Agricola Olere, located in the Apulia region (Ostuni, BR) as previously reported [21]. The leaves were dried in an oven at 40 °C to constant dry weight to ensure stable moisture content (90–93 %), and then finely ground using a Grindomix GM 200 knife mill (Retsch, Haan, Germany). The ground samples collected from June to August were homogenously pooled and then sieved to standardize particle size distribution between 300 and 600 µm. The resulting CSL material was stored under vacuum until use. All extraction yields and quantitative data were expressed on a dry weight (DW) basis.

### 2.3 Exhaustive extraction

Exhaustive extraction of CSL was achieved by ultrasound-assisted solid–liquid extraction in a thermostatic ultrasounds bath (Labsonic LBS2, Treviglio, Italy). The extraction was performed using aqueous ethanol (70 % v/v) and a solid–liquid ratio of 50 g L^–1^, following the previously reported procedure [21]. Filtered supernatants (Whatman No. 1 filter paper) from three extraction cycles were pooled, and ethanol was removed under vacuum at 40 °C using a rotary evaporator. All extractions were conducted in triplicates. The resulting aqueous residues were then freeze-dried (freeze dryer Alpha 1–2 LD, Christ, Germany) to determine the extraction yield (EY), expressed as g extract per 100 g dry leaves (g 100 g^−1^ dry leaves, mean ± standard deviation (SD)).

### 2.4 Conventional solid–liquid extractions

Magnetic stirring-assisted maceration (SLE) and Soxhlet extraction (So) were employed as conventional solid–liquid extraction techniques using aqueous ethanol (70 % v/v) as extraction solvent. For Soxhlet extraction, approximately 5  g of CSL was placed in a cellulose thimble and extracted in a Soxhlet extractor with 100  mL of solvent under reflux for 8  h. For maceration, the same amounts of sample and solvent were stirred at room temperature for 24 h. Both extraction techniques were conducted in triplicates. After extraction and filtration, the extracts were treated as detailed in Section 2.3.

### 2.5 Pressurized hot water extraction (PHWE)

PHWE was performed with an ASE 350 system (ThermoFisher Scientific, Milano, Italy) in static extraction mode using 34 mL stainless steel cells and 250  mL collection bottles. Extraction cells, equipped with stainless steel frits and a cellulose filter at the bottom, were loaded with sample mixed with 4 mm glass beads. The fixed instrumental parameters were set as follows: distilled water as extraction solvent, pressure of 100 bar, purge of 40 sec, rinse equal to 50 % cell volume, and cell preheating deactivated. For preliminary experiments, a single extraction cycle with a static time of 10 min was applied. Under optimal conditions, PHWE was performed at a temperature of 98 °C with three cycles, each with a static time of 5 min, loading 1.5 g of matrix into 34 mL cells.

After cooling, PHWE extracts were diluted to a final volume of 60 mL with water. An aliquot of 1 mL was directly used for UHPLC and spectrophotometric analyses, while the remaining volume was freeze-dried to determine the extraction yield (EY).

### 2.6 Ultrasound-assisted extraction using probe system (probe-UAE)

Probe-UAE was conducted using an ultrasonic processor UP400St (Hielscher Ultrasonics GmbH, Teltow, Germany) equipped with a titanium sonotrode H3 (3 mm diameter, for volume from 5 to 200 mL) at a fixed working frequency of 24 kHz. CSL samples were mixed with 20 mL of solvent in a 50 mL centrifuge tube and extracted by immersing the probe 1 cm deep into the suspension at 30 °C. The temperature was continuously monitored using a thermocouple and controlled by a circulating water bath. For preliminary experiments, a solid–liquid ratio of 25 g L^–1^ and an extraction time of 30 min were used to assess the influence of amplitude, duty cycle, extraction solvent, and extraction kinetics. Subsequently, the operational parameters were set to an amplitude of 60 % and a duty cycle of 50 %. Under optimal conditions, UAE was performed using aqueous ethanol 52 % v/v, with a solid–liquid ratio of 100 g L^–1^ and an extraction time of 20 min.

UAE extracts were filtered through a Whatman No. 1 filter paper. An aliquot of 1 mL was processed for subsequent analyses, and the remaining extract was treated as described in Section 2.3.

### 2.7 Experimental design and optimization

PHWE and probe-UAE conditions were optimized by Response Surface Design using a Box-Behnken design quadratic model (19 degrees of freedom), consisting of two block replicates of 15 randomized experimental runs and three center points for block. The experimental design and optimization of operational conditions for each extraction process were performed using Statgraphics Centurion 18 software, provided by Statgraphics Technologies (Adalta snc, Arezzo, Italy).

The independent experimental factors considered for each extraction method and their levels (– 1, 0, and + 1) are reported in Table 1. Minimum and maximum levels of the independent factors were selected based on preliminary experiments. Extraction efficiency (EE, mg g^−1^ leaf) and extract purity (P, g 100 g^−1^ extract) of the main CSL compounds (Sil, Myr, and TeGG) were designated as response variables to be maximized. Gallic acid (GA) and digalloylglucoses (diGGs) contents (mg g^−1^ leaf) were monitored as degradation indicators to be minimized in PHWE and probe-UAE optimization, respectively. Furthermore, extraction yield (EY, g extract 100 g^−1^ leaf) and total phenolic content (TPC, mg GAE g^−1^ leaf) were considered as response variables. The experimental conditions for both designs and the corresponding response variable values are listed in Table S1 (PHWE) and S2 (probe-UAE) of the Supplementary data.

**Table 1:** Independent factors and their levels for PHWE and probe-UAE.

**Extraction method**	**Independent factors**	**Units**	**Level**		
			**− 1**	**0**	**1**
**PHWE**^a^	A. Temperature	°C	60	100	140
	B. Static time	min	5	8	11
	C. cycle	n	1	2	3
**UAE**^b^	A. % EtOH	v/v	30	50	70
	B. Extraction time	min	10	15	20
	C. solid–liquid ratio (SLR)	g L^−1^	20	60	100

a extraction solvent, water; pressure, 100 bar; purge, 40 sec; rinse, 50 % cell volume.

b solvent volume, 20 mL; frequency, 24 kHz; amplitude, 60 %; duty cycle, 50 %.

A second-order polynomial equation was used to fit each dependent variable (Y) as a function of the independent factors (A-C). Mathematical models of the estimated response surfaces were defined after removing the non-significant factors and two-factor interactions identified by ANOVA (p > 0.05). Model adequacy was verified using the lack-of-fit test, which assesses whether the model appropriately describes the observed data. P-values greater than 0.05 indicate that the fitted model adequately represents the data. Model quality was evaluated through the regression coefficients R^2^ and adjusted R^2^, which indicate the proportion of variability in response variables explained by the model. Optimal R^2^ and adjusted R^2^ values range between 0.8 and 1. Subsequently, simultaneous optimization of independent factors was conducted using the desirability function aiming to maximize EEs and Ps of main CSL compounds while minimizing GA and diGGs contents. The suggested optimal conditions were experimentally validated by comparing predicted values of response variables with the experimental data of six extraction replicates (95 % confidence interval).

### 2.8 UHPLC-UV quantitative analysis

Quantitative analysis of CSL extracts was performed using a Vanquish Flex UHPLC system interfaced to a Diode Array Detector FG and an Orbitrap Exploris 120 mass spectrometer, equipped with a heated electrospray ionization source, HESI-II (ThermoFisher Scientific, Milano, Italy). UHPLC separation was performed using a previously developed method [21]. Briefly, the chromatographic separation was achieved with a Kinetex C18 column (2.1 × 100 mm, 2.6 μm; Phenomenex, Bologna, Italy), protected by a C18 Guard Cartridge (2.1 mm I.D.) and operated at 30 °C. The binary gradient consisted of water (A) and MeCN (B), both containing 0.1 % formic acid, at a flow rate of 500 µL min^−1^. Target analytes were quantified at 280 nm for galloyl derivatives and 350 nm for flavonols.

The levels of the main CSL compounds were determined by a validated UHPLC-UV quantification method [21], using external standard calibration. GA, TeGG, Sil and Myr were used as reference standards. The levels of diGGs, for which no reference standards were available, were estimated using the calibration curves of GA. Quantitative results were expressed as g per 100 g of extracts (g 100 g^−1^ extract, mean ± SD) and mg per g of dry leaf (mg g^−1^ DM, mean ± SD).

### 2.9 UHPLC-HRMS analysis of degradation products

UHPLC-HRMS analysis was performed using the same chromatographic condition described in Section 2.8 and the mass acquisition parameters reported in [21]. Extracted ion chromatograms of pyrogallol ([M − H]^−^ at *m*/*z* 125.0244) and 5-hydroxymethylfurfual ([M + H]^+^ at *m*/*z* 127.0390) with a mass selection window of 3 ppm were used for their quantitation. The concentration levels of pyrogallol were estimated using solvent calibration curves (0.5-20μg mL^−1^).

### 2.10 Determination of the total phenolic content (TPC) and antioxidant capacity (AOC)

TPC and AOC of CSL extracts were determined using the Folin-Ciocalteu and ABTS scavenging assays, respectively, as reported in our previous study [21]. Assays were conducted using a Varioskan LUX multimode microplate reader (Thermo Fisher Scientific). TPC was estimated from the calibration curve of GA (range 5–200μg mL^−1^) and the data were expressed as GA equivalents per g of leaf (mg GAE g^−1^ leaf, mean ± SD). AOCs were expressed as Trolox equivalent antioxidant capacity (TEAC) per g of extract (mmol TE g^−1^, mean ± SD). Trolox calibration curves (2.5–25 µM, well concentrations) were used to calculate AOCs.

### 2.11 *In vitro* α-glucosidase (α-GLU) inhibitory assay

The inhibitory activity of α-GLU was evaluated according to the procedure reported by Serio et al., [21]. Acarbose (0.05–1 mM, well concentration) and quercetin (2–50 µM, well concentration) were used as positive controls. Phosphate buffer (50 mM, pH = 6.8) was used for dilutions of the extracts. Enzymatic activity was evaluated by measuring absorbance at 405 nm with a Varioskan LUX microplate reader. Results were expressed as IC_50_ (concentration of inhibitor that causes 50 % enzyme inhibition, μg mL^−1^ ± SD).

### 2.12 Cell cultures and MTT assay

Human hepatocellular carcinoma cells HepG2 were purchased from ATCC (American Type Culture Collection) and cultured in DMEM (Dulbecco’s Modified Eagle Medium) low glucose, with 10 % of FBS (Fetal Bovine Serum) and 1 % Pen-Strep. Cell lines were grown at 37 °C in 5 % CO_2_ humidified atmosphere. Dilutions were prepared in culture medium immediately prior to use. In all experiments, the final concentration of DMSO did not exceed 0.5 % (v/v).

HepG2 cells were seeded into 96-well plates at a density of 1 x 10^4^ cells/well in four replicates and exposed to increasing concentration (5–300μg mL^−1^) of the extracts for 24 h. Cell viability was assessed using the colorimetric MTT assay. To this end, after 24 h, the culture medium was removed, and 50 μL of MTT (0.5 mg mL^−1^ PBS) was added to each well and incubated for 4 h at 37 °C. Subsequently, the medium was discarded, and the formazan crystals were solubilized with 200 μL of DMSO. MTT conversion to formazan by metabolically viable cells was monitored at an optical density of 540 nm. Results (percentage of cell viability) are expressed as mean ± SD of three independent experiments.

### 2.13 Statistical analysis

Results are reported as mean ± standard deviation (SD) from three or six replicates. The statistical significance of quantitative data, TPC, TEAC and IC_50_ values were evaluated through one-way analysis of variance (ANOVA). A significance level of p < 0.05 was adopted, and Tukey's honestly significant difference (HSD) test was performed to determine pairwise differences between means. Statistical analyses were carried out using Statgraphics Centurion 18 software (Statgraphics Technologies), while IC_50_ values were calculated using GraphPad Prism 10.

## 3 Results and discussion

### 3.1 Pressurized hot water extraction (PHWE)

In PHWE, high temperatures and pressures significantly modify the physicochemical properties of water, enhancing analyte solubility and mass transfer. Along with the reduction of viscosity and surface tension, the dielectric constant of water decreases under PHWE, achieving a polarity comparable to that of alcoholic solvents at atmospheric conditions. Therefore, water polarity can be tuned by adjusting temperature and pressure, enabling the extraction of compounds with different polarities [11,13].

PHWE efficiency is primarily affected by temperature, pressure and extraction time. Additional factors, such as particle size, solid–liquid ratio, dispersant agent, and modifier, may also impact the extraction performance [11,13]. In this study, pure water was selected as the extraction solvent to ensure process sustainability and avoid costly organic solvent evaporation or recycling.

#### 3.1.1 Preliminary PHWE experiments

Preliminary experiments were conducted to define specific technical parameters, reduce the number of independent factors for chemometric analysis, and establish their experimental domain. Extractions were performed in triplicate at 100 bar, using a single cycle with a static time of 10 min. PHWE extracts were analyzed by UHPLC-UV to monitor the quali-quantitative profile of CSL extracts. The effect of temperature on the recovery and stability of target compounds (Sil, TeGG and Myr) was initially assessed. A range of 60–175 °C was explored to determine the maximum operating threshold. Within this temperature range, the dielectric constant of water decreases by about 40 % [23], enhancing its solvent capacity and the solubility of medium-polar compounds. However, high temperatures may also induce the degradation of thermolabile compounds and promote the Maillard reaction, potentially affecting extract quality and bioactivity [5,13,15,24].

**Fig. 1:**
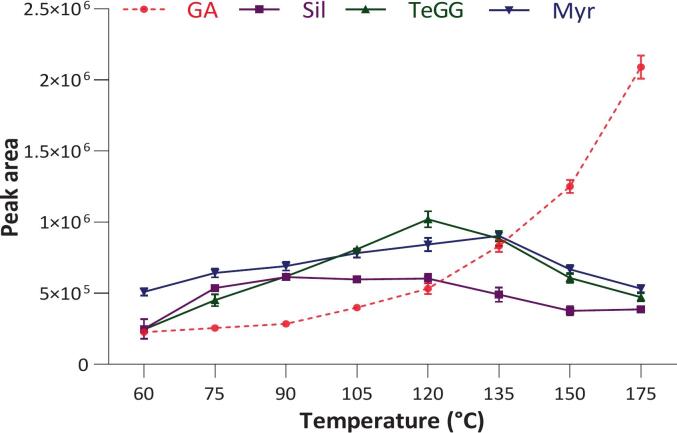
Temperature-trend of main CSL compounds (Sil, TeGG and Myr) and hydrolysis indicator (GA). Extractions were carried out in triplicate in the following PHWE conditions: extraction solvent, water; pressure, 100 bar; static time, 10 min (1 cycle); purge, 40 sec; rinse, 50 % cell volume.

Temperature-dependent trends (Fig. 1) indicated a positive correlation between Myr content and temperature up to 135 °C. In contrast, TeGG and Sil recoveries declined significantly beyond 120 °C, likely due to the hydrolysis of gallate ester bonds, as supported by the concurrent increase in gallic acid (GA) levels above 90 °C. A similar temperature-dependent hydrolytic effect on galloylated compounds were reported in pistachio hulls [25], chestnut shells [26] and winery by-products [27] subjected to SWE at high temperatures. In these studies, the release of gallic acid was attributed to the hydrolysis of hydrolyzable tannins and galloylated procyanidins.

Furthermore, at elevated temperatures, gallic acid undergoes decarboxylation, forming pyrogallol as a primary degradation product [26]. Significant amounts of pyrogallol were produced from 135 °C onwards, indicating the onset of thermal decomposition. Regarding 5-hydroxymethylfurfual (HMF), a marker of Maillard and caramelization reactions, only trace levels were detected above 150 °C. These data agree with previous studies reporting HMF formation in SWE extracts at temperatures above 130–150 °C [15,25,28,29]. Based on these findings, 140 °C was defined as the maximum temperature for PHWE to prevent the formation of degradation artefacts.

Subsequently, the influence of dispersant type (diatomaceous earth, sand, glass beads) and matrix amount (1–3 g) was investigated. No significant interaction between analytes and dispersants were observed; however, diatomaceous earths produced extracts containing suspended solid material, likely released from dispersant under the applied conditions. Therefore, glass beads were selected due to their reusability. Concerning matrix amount, 1.5 g was established as the optimal quantity to ensure efficient analyte recovery.

#### 3.1.2 Optimization of PHWE by RSM

Insights from preliminary experiments highlighted temperature as a critical factor influencing CSL extract quality, which is primarily determined by the content of active compounds and the absence of denatured molecules or extraction artefacts [5,30]. Moreover, extraction time also plays a crucial role in the stability of labile compounds, as prolonged exposure to high temperatures can further promote degradation [11,13,15]. Therefore, simultaneous optimization of temperature and extraction time in PHWE is essential to balance extraction efficiency and bioactive compound stability.

A RSM based on a Box-Behnken Design (BBD) was applied to evaluate the effects of temperature (A), static time (B), and the number of cycles (C) (Table 1) on PHWE effectiveness, as well as their optimization to maximize desired outcomes. Optimization focused on maximizing the extraction efficiency (EE, mg g–1 leaf) and extract content (P, g 100 g–1 extract) of target compounds (Sil, TeGG and Myr), evaluated as key response variables. Furthermore, GA levels were monitored as an indicator of hydrolytic degradation of TeGG and Sil and considered a response variable to be minimized. Extraction yield (EY) and total phenolic content (TPC), common responses in experimental designs for phenolic extraction, were also considered as response variables. The results for all these variables, obtained under different BBD experimental conditions, are reported in Table S1. The highest levels of Sil, TeGG and Myr were consistent with previously reported data for *C. siliqua* leaves of the Amele variety (3.9–5.6, 5.2–6.9 and 2.7–3.3 g 100 g^−1^ extract, respectively for Sil, TeGG and Myr) [21]. These results indicate that PHWE can achieve complete recovery of target analytes from the matrix.

The effect of experimental factors A-C, their interactions, and statistical significance were determined by ANOVA (Table S3). Pareto charts (Fig. S1) indicated that temperature (A) and cycle number (C) were the most significant factors for all target responses. In contrast, only EY, TPC, Sil and GA were directly affected by static time (B) (Fig. S1). Temperature, its quadratic term (AA) and interactions with static time (AB) and cycle number (AC), exhibited opposing effects on Sil and TeGG compared to GA (Fig. S1).

The quadratic models (Table S4), containing only significant terms (p < 0.05), were highly significant (p < 0.001) and accurate (p-values of lack-of-fit > 0.05) for all variable responses. The adjusted R^2^ values of 88–98 % indicated a robust model predictability (Table 3S).

**Fig. 2:**
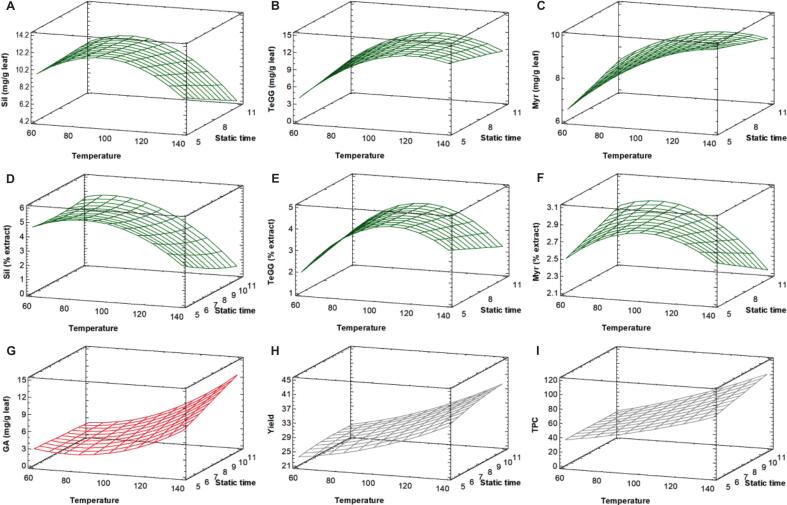
Response surface plots of extraction efficiency of Sil (A), TeGG (B), Myr (C) and GA (G); extract content of Sil (D), TeGG (E) and Myr (F); extraction yield (H); and TPC (I) as a function of temperature and static time for PHWE of bioactive compounds from *Ceratonia siliqua* leaves. The cycle number was kept constant at the center point (n = 2). Only statistically significant effects are considered.

The combined significant effects of PHWE factors (A–C) (Fig. 2 and Fig. S2) showed that temperature exerted a pronounced influence on all response variables. Higher temperatures enhanced PHWE efficiency by improving water diffusivity and solubility. However, EE of Sil and TeGG (Fig. 2A–B) decreases beyond 90 and 120 °C (Table S4), respectively. Conversely, GA levels rose with increasing temperature (Fig. 2G), confirming that Sil and TeGG are susceptible to hydrolysis of galloyl ester bonds at higher temperatures. A longer static time further compromised the stability of Sil, resulting in lower recovery and a simultaneous rise in GA (Fig. 2A and G), whereas splitting extraction time into multiple cycles (Fig. S2) helped mitigate Sil hydrolysis. This trend suggests that milder cyclic PHWE conditions better preserve the naturalness of PHWE extracts and potentially their functionality.

The extract content of target compounds was also markedly affected by temperature, both independently and in combination with static time (AB) and cycle number (AC), showing a decline beyond 65–100 °C (Fig. 2D–F and Table S4). This behaviour likely results from the co-extraction of matrix components under more intensive PHWE conditions, which reduces the target compound content in the extract. Indeed, the response surface for EY (Fig. 2H and Fig. S2H) showed an upward trend with increasing temperature, static time and number of cycles. Thus, moderate PHWE conditions also favor a more selective recovery of bioactive compounds, yielding extracts richer in bioactive compounds. Overall, these findings highlight that temperature and its interaction with static time and cycle number, is a key parameter in PHWE for thermolabile compounds, as it influences both extraction efficiency and hydrolysis of Sil and TeGG. Therefore, a multiple response analysis was performed to simultaneously optimize PHWE conditions using the desirability function derived from the BBD (Fig. S3). The optimal PHWE conditions were identified as a temperature of 98 °C and three extraction cycles of 5 min (desirability of 0.90). These conditions were experimentally validated, and all results fell within the predicted optimal range (Table S1), confirming the accuracy of the prediction model.

**Fig. 3:**
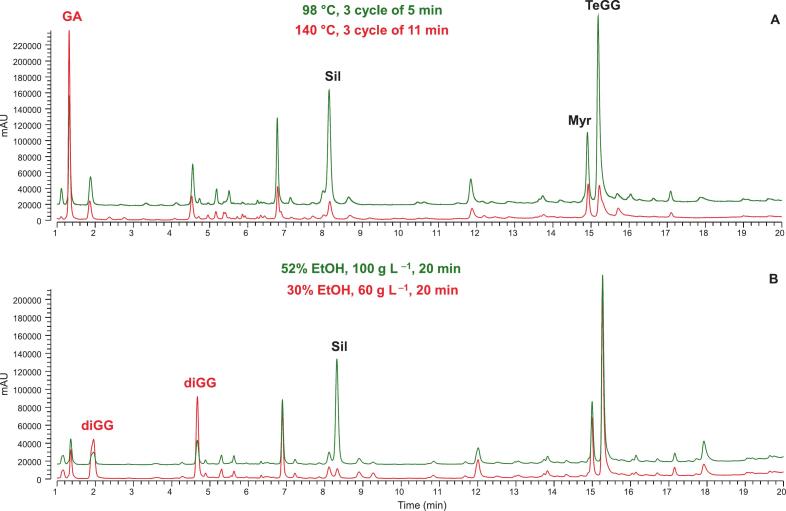
UHPLC-UV profiles at 280 nm of PHWE (A) and probe-UAE (B) extracts obtained under optimal conditions (green line) or based on extraction yield and TPC (red line).

If PHWE optimization prioritized TPC and EY, predicted optimal PHWE conditions corresponded to the maximum values of the considered factors (140 °C, three cycles of 11 min). However, as shown in PHWE extract profiles (Fig. 3A) for the two different optimized conditions, the more intense conditions led to extensive hydrolysis of Sil and TeGG. Moreover, the degradation product pyrogallol was formed under the PHWE conditions optimized based on TPC and EY (6 mg g^−1^ extract). This underscores that, although TPC and extraction yield are commonly used to optimize phenolic compound extraction in experimental designs, relying solely on these responses does not guarantee the extract quality, as demonstrated by various studies [26,28,31]. In this context, specific output responses, such as chemical profile, target compound levels and degradation indicators, provide a more reliable evaluation of extraction performance when the goal is to obtain high-quality extracts.

### 3.2 Ultrasound-Assisted extraction using probe system (probe-UAE)

Unlike PHWE, UAE is a non-thermal extraction technique widely employed to enhance the recovery of bioactive compounds from plant matrices within a shorter extraction time. UAE enables efficient and rapid extraction by leveraging the acoustic cavitation effect, which disrupts cell walls enhancing solvent penetration into the matrix and promoting mass transfer [16,18].

The most significant factors influencing UAE efficiency include the solvent type, extraction time and solid–liquid ratio. Additionally, ultrasonic parameters such as system type, frequency, power and duty cycle also impact UAE performance [9,16,17]. In this study, a probe-based system was selected over a bath system due to its higher ultrasonic intensity, reproducibility, and suitability for large-scale applications. A constant low frequency (24 kHz) was employed as various studies indicate that lower frequencies generate fewer but larger cavitation bubbles, thereby enhancing the cavitation effect [9,16,17].

Regarding the extraction solvent, water and aqueous ethanol mixtures were evaluated. Ethanol–water mixtures are widely used for extracting phenolic compounds from plant matrices due to their high affinity for these bioactive compounds [14,16,17]. Moreover, ethanol is widely used for its affordability, renewable origin, and classification as a GRAS solvent.

#### 3.2.1 Preliminary probe-UAE experiments

**Fig. 4:**
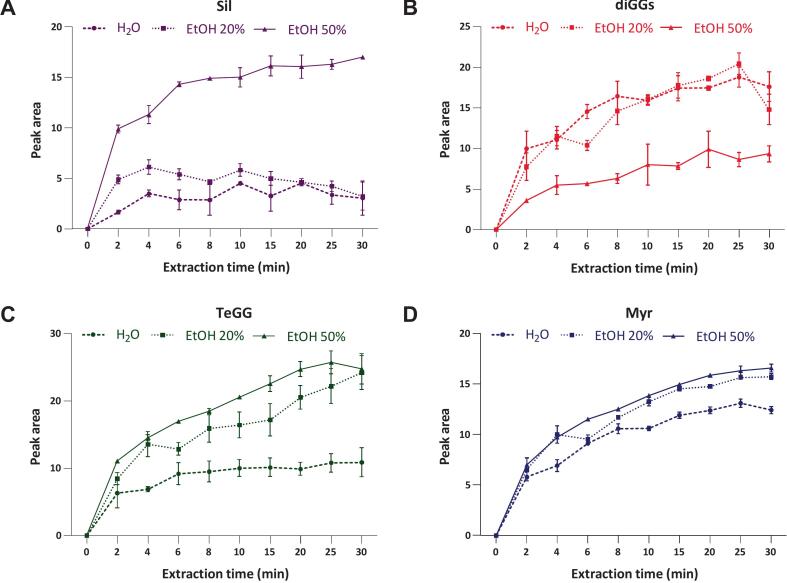
Extraction kinetics of main CSL compounds (A, Sil; C, TeGG; D, Myr) and Sil degradation indicator (B, diGGs). Extractions were carried out in triplicate in the following UAE conditions: temperature, 30 °C; solid–liquid ratio (SLR), 25 g L^–1^; frequency, 24 kHz; amplitude, 100 %; duty cycle, 50 %.

Various experiments were preliminarily carried out to define specific technical parameters and operational ranges of key UAE factors (solvent, time and power) for subsequent RSM optimization. Extractions were carried out in triplicate at 30 °C with a solid–liquid ratio (SLR) of 25 g L^–1^. Kinetic curves of CSL compounds were obtained by UHPLC-UV analysis of UAE extracts collected at different time intervals (2–30 min) (Fig. 4).

Initially, water and two aqueous ethanol mixtures (EtOH 20 % and 50 %, v/v) were tested as extraction solvent. EEs of target CSL compounds improved with increasing ethanol content (Fig. 4). Notably, only 50 % aqueous ethanol provided acceptable and reproducible Sil recovery (Fig. 4A). Meanwhile, CSL profiles revealed an inverse trend in diGG levels as function of % EtOH (Fig. 4B). These findings suggest that, in aqueous medium, Sil may undergo conversion to diGG following loss of its pyranone moiety. The involvement of reactive radicals generated during cavitation bubble implosion [16,17] was ruled out by processing CSL extract dissolved in water under probe-UAE conditions. It is more likely that Sil hydrolysis was triggered by vacuolar processing enzyme released upon cell disruption [32]. Based on these observations, 30 % ethanol was selected as the starting point for defining optimal working conditions. Extraction kinetics (Fig. 4) also indicated that the diffusion-controlled period, during which most soluble solute was already recovered, began around 20 min. Consequently, the maximum extraction time was set at 20 min.

Regarding ultrasonic power, different amplitudes (30, 60 and 100 %) and duty cycle percentages (50 and 100 %) were evaluated to maximize extraction efficiency. The results indicated no relevant variations in the recoveries of Sil, TeGG and Myr across the tested ranges (data not shown). Thus, amplitude and duty cycle were set at of 60 % and 50 %, respectively, to reduce energy consumption and prevent system overheating.

#### 3.2.2 Optimization of probe-UAE by RSM

Following the approach used for PHWE, probe-UAE conditions were optimized by RSM to evaluate the individual and combined effects of solvent type (A), extraction time (B) and SLR (C) (Table 1). The same response variables as PHWE (EE and P of Sil, TeGG and Myr) were considered, along with ethanol volume (L per kg matrix) to assess extraction sustainability. Additionally, diGG levels were monitored as indicator of Sil degradation. The experimental results obtained under different BBD conditions are reported in Table S2. Similarly to PHWE, UAE proved effective in achieving exhaustive extraction of bioactive CSL compounds [21], as evidenced by the maximum levels of Sil, TeGG, and Myr in the extracts.

The effect of factors A-C on response variables, determined by ANOVA analysis (Table S5 and Fig. S4), indicated that all three factors, as well as the quadratic terms of % EtOH (AA) and SLR (CC), significantly affected EY and EEs of TeGG and Myr (p < 0.001), consistent with the consolidate literature data [[16], [17], [18]]. However, EEs of Sil and diGGs were strongly influenced by EtOH percentage and its quadratic term (A and AA), but with opposite trends (Table S5 and Fig. S4). Extraction time (B and AB) also showed significant, though less pronounced, effect on these responses. Regarding the content of target compounds in the extracts, it was negatively affected by the quadratic term of % EtOH (AA, p < 0.05) (Fig. S4).

**Fig. 5:**
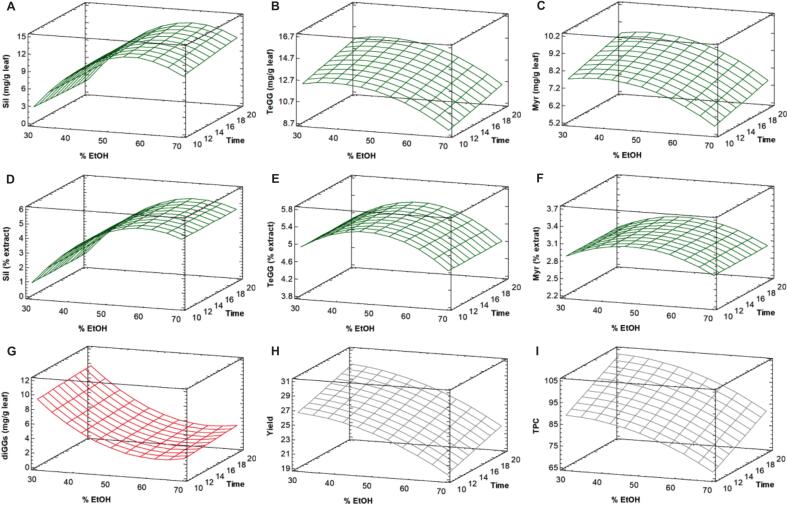
Response surface plots of extraction efficiency of Sil (A), TeGG (B), Myr (C) and GA (G); extract content of Sil (D), TeGG (E) and Myr (F); extraction yield (H); and TPC (I) as a function of % EtOH and extraction time for probe-UAE of bioactive compounds from *Ceratonia siliqua* leaves. The solid–liquid ratio (SLR) was kept constant at the center point (60 g L^−1^). Only statistically significant effects are considered.

The regression model equations (Table S6), resulting from the removal of statistically no significant terms, were significant (p < 0.0002) and accurate (p-values of lack-of-fit > 0.05) (Table S5). The combined effects of UAE factors, as depicted in the response surface plots (Fig. 5 and Fig. S5), confirmed that %EtOH was the dominant factors influencing all response variables. Specifically, EE of Sil (Fig. 5A and Fig. S5A) primarily varied as a function of %EtOH, reaching maximum values at 58 % EtOH (Table S6), while extraction time and SLR had negligible effects. The opposite effects of %EtOH on diGGs corroborate the hypothesis of Sil by enzymatic hydrolysis into diGGs at EtOH percentages below 50 %. Above this threshold, enzymatic activity likely ceases due to denaturation induced by the higher organic solvent content. Indeed, most natural enzymes are easily inactivated in the presence of organic solvents [33]. Unlike Sil, the recoveries of TeGG (Fig. 5B and Fig. S5B) and Myr (Fig. 5C and Fig. S5C), as well as EY (Fig. 5H and Fig. S5H) and TPC (Fig. 5I and Fig. S5I), were significantly influenced by all three independent factors. Extraction time linearly improved these response variables, according to preliminary experiments (Section 3.2.1). However, above 38 % EtOH (Table S6), EEs of TeGG and Myr declined due to the decrease in the solvent’s dielectric constant.

Regarding extract composition, % EtOH was the only significant factor affecting the extract contents of Sil, TeGG, and Myr (Fig. 5D–F and Fig. S5D–F). These responses peaked at an intermediate % EtOH (48–60 %) before declining. These results highlight that solvent selection in UAE depends primarily on the solubility and polarity of target compounds. In this case, the main CSL compounds were more effectively extracted using a low-medium ethanol content. Similar trends have been reported for other plant matrices [[34], [35], [36]], where aqueous ethanol mixtures enhance improve phenolic recovery up to a certain %EtOH, after which high ethanol contents may cause plant tissue dehydration, protein denaturation, or the solubilization of additional matrix components due to the stronger solvent power [16]. Overall, % EtOH emerged as the dominant factor influencing both probe-UAE efficiency and extract quality. Sil stability and extract enrichment were found to be the responses most affected by this factor, with consequent impacts on the quality and bioactivity of the final extract.

The simultaneous optimization of the three studied factors by multiple response analysis identified an aqueous EtOH solution of 52 % v/v, a SLR of 100 g L^–1^ and an extraction time of 20 min (desirability of 0.83) as the optimal UAE conditions (Fig. S6) to maximize CSL compound recovery and produce enriched extracts, while preventing Sil hydrolysis. Experimental validation confirmed that all responses remained within the 95 % confidence interval of predicted values (Table S2), demonstrating the robustness of the established models for desired outcomes.

Even for UAE, the optimal conditions predicted solely based on TPC and EY (EtOH 30 % v/v, 65 g L^–1^, 20 min, desirability of 0.79) led to degraded extracts (Fig. 3B). UAE extract profiles indicated that lower % EtOH promoted Sil hydrolysis to diGGs. This further emphasizes the importance of optimizing extraction conditions based on specific target compound levels, using chromatographic methods rather than less selective spectrophotometric methods, to ensure high quality extracts containing intact bioactive compounds [[34], [35], [36]].

### 3.3 PHWE and probe-UAE performance vs conventional methods

Following the optimization studies, the performance of PHWE and probe-UAE in extracting bioactive compounds from CSL was evaluated and compared with two conventional extraction methods, such as Soxhlet extraction (So) and magnetic stirring-assisted maceration (SLE).

As a first step, product recovery (amount of bioactive compounds recovered from the matrix) of PHWE and probe-UAE, health promoting potential (antioxidant capacity and α-glucosidase inhibition) and *in vitro* cytotoxicity (viability of and hepatic (HepG2) cells by MTT assay) of extracts were determined and benchmarked against exhaustive extraction [21]. Both optimized techniques achieved complete recovery of the main bioactive compounds from CSL (88–108 %) with no statistically significant differences (p > 0.05) (Table 2). PHWE and UAE extracts retained the *in vitro* bioactivities (Table 2) observed in the exhaustive CSL extract (AOC, 4.6 ± 0.2 mmol TE g^−1^; IC_50 α-GLU_, 0.45 ± 0.03μg mL^−1^). This was consistent with the composition data of PHWE and UAE extracts (Tables S1 and S2), which showed no significant differences from the exhaustive extract in terms of Sil, TeGG, and Myr content (5.5, 5.2 and 3.3 g 100^–1^ extract, respectively), confirming that CSL bioactivity is primarily attributed to its main compounds, as previously demonstrated [21]. Furthermore, neither PHWE nor UAE extracts exhibited significant cytotoxicity at the highest concentrations (300 µg mL^−1^) on HepG2 viability (Fig. S7), suggesting a favourable safety profile for both extracts. These results demonstrate that both optimized techniques not only ensure exhaustive recovery of the target compounds but also preserve the biological functionality of CSL, which are critical for nutraceutical and functional food applications.

**Table 2:** Extraction performance of optimized PHWE and probe-UAE compared with conventional techniques. a

	**PHWE**	**probe-UAE**	**Soxhlet**	**Maceration**
Extract yield (g 100 g^−1^ leaf)	29.6 ± 0.7^a^	27.8 ± 0.6^b^	20.3 ± 0.2^c^	16.0 ± 0.8 ^d^
Sil recovery (%)	87.6 ± 1.7^a^	89.6 ± 4.8^a^	68.0 ± 6.0^b^	55.4 ± 3.0^c^
TeGG recovery (%)	103.3 ± 2.6^a^	103.5 ± 4.5^a^	78.0 ± 5.0^b^	71.4 ± 3.8^b^
Myr recovery (%)	107.9 ± 3.0^a^	101.9 ± 2.8^a^	74.8 ± 5.3^b^	57.2 ± 3.0^c^
ABTS (mmol TE g^−1^ extract)	4.7 ± 0.3^a^	4.8 ± 0.4^a^	3.3 ± 0.2^b^	5.1 ± 0.3^a^
α-glucosidase (IC_50_, µg mL^−1^)	0.47 ± 0.07^a^	0.44 ± 0.06^a^	0.48 ± 0.06^a^	0.46 ± 0.04^a^
**Green extraction principle**	**PHWE**	**probe-UAE**	**Soxhlet**	**Maceration**
1. Raw material (g g^−1^ extract)	3.4	3.6	4.9	6.2
2. Ethanol volume (L g^−1^ extract)	0.00	0.02	0.07	0.09
3. Energy consumption (Wh per extraction cycle)	125	40	1200	400
4. Waste (g g^−1^ extract)	3.1	18.3	59.8	75.9
5. Extraction time (h per extraction cycle)	0.25	0.3	8	24
6. Product recovery (%)	98.3	97.7	73.4	61.8

a Different superscript letters in the same row indicate significantly different values (p ≤ 0.05 by a Tukey's HSD test).

PHWE and probe-UAE were specifically designed and optimized for the sustainable recovery of target CSL compounds. Therefore, their greenness performance was assessed according to the six principles of green extraction [5] and compared with So and SLE. In line with previous studies [[37], [38], [39]], parameters were considered included raw material consumption (Principle 1, g of matrix per g of extract), organic solvent use (Principle 2, EtOH volume per g of extract), energy consumption (Principle 3, kWh per extraction), waste generation (Principle 4, g of spent material and solvent per g of extract), extraction time (Principle 5, h per extraction), and recovery efficiency (Principle 6, % of target compounds recovered) (Table 2).

**Fig. 6:**
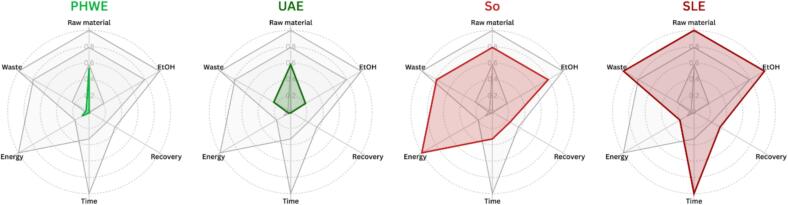
Process assessment of PHWE, probe-UAE and conventional methods according to the six green extraction principles. Data are normalized on a 0–1 scale. Lower values indicate better performance.

The comparative analysis is presented in Fig. 6, where values closer to the center indicate better compliance. PHWE displayed the most favorable profile (area = 0.012), closely followed by probe-UAE (area = 0.117), whereas So and SLE were markedly less efficient (area = 1.147 and 1.360, respectively). Both techniques significantly outperformed So and SLE across all evaluated parameters.

Quantitative comparisons (Table 2) illustrate the environmental advantages: both techniques required lower amounts of raw material (27–45 %) to yield 1 g of extract, and PHWE required exclusively water, while probe-UAE reduced ethanol use 4–5-fold relative to conventional methods. This contributed to a lower waste output, in terms of solvent and spent residue, leading to a 95 % reduction in waste generation for PHWE compared to So and SLE. Moreover, So and SLE failed to achieve full recovery of the target compounds (Table 2), leaving 27–38 % of the bioactive compounds in partially exhausted residues. Another clear advantage of PHWE and probe-UAE over conventional methods was the markedly reduced extraction times, achieving a 24–32 and 73–96 times faster extraction than So and SLE, respectively. These shorter extraction times strongly impact the energy demands. Probe-UAE emerged as the most energy-efficient option, with energy consumption 30-, 6- and 3-fold lower than So, SLE and PHWE, respectively (Table 2). Despite PHWE is the most energy-intensive, it still required 10 times less energy than So due to faster extraction duration.

These findings highlight the environmental and technical benefits of PHWE and probe-UAE over conventional extraction methods. Both approaches enable scalable, solvent-saving and energy-efficient processes, aligning with industrial sustainability requirements. Such improvements directly address the goal of study of sustainable valorization of CSL, offering sustainable and more efficient alternatives to conventional extraction methods that maximize extraction yield while preserving extract quality and functionality.

## 4 Conclusion

This study demonstrated the successful application of PHWE and probe-UAE as effective and sustainable strategies for the recovery of valuable compounds from carob leaves (CSL), an underutilized plant biomass with promising antioxidant and hypoglycaemic potential. By integrating RSM with UHPLC profiling, the processing parameters influencing extraction efficiency and stability of target compounds were systematically elucidated. Temperature and ethanol concentration emerged as the most critical parameters affecting the effectiveness of PHWE and probe-UAE, respectively. The optimized extraction conditions, tailored to maximize the recovery of Sil, TeGG and Myr while minimizing their degradation, enabled an exhaustive extraction of CSL bioactive compounds yielding safe and high-quality extracts and preserved functional properties.

In accordance with green extraction principles, both PHWE and probe-UAE exemplify process intensification compared to conventional extraction methods such as Soxhlet and maceration. These techniques demonstrated excellent greenness with minimal solvent and energy demands, shorter extraction time, higher recovery yields, and reduced waste generation. Among the two, probe-UAE offers advantages in energy efficiency, operational simplicity and low investment cost. However, it requires additional post-extraction treatments, which are translated in longer and more labour-intensive processes. In contrast, although PHWE involves the use of specialized and expensive equipment, it eliminates organic solvents entirely, producing aqueous extracts suitable for direct use or formulation. Moreover, water is recognized as the most environmentally friendly solvent and PHWE represents a significant step towards sustainability for the recovery of valuable compounds [5,14]. These complementary features highlight their potential for integration into industrial-scale extraction processes aligned with green chemistry and circular economy principles.

Overall, this study provides a scientific basis for the sustainable valorization of plant matrices and agricultural co-products as source of high-value compounds for use as functional ingredients in various food products. Although both techniques have shown promising results at laboratory scale, further research is required to support their transition to industrial applications. In particular, pilot-scale validation and comprehensive assessments of environmental impact and economic feasibility are essential to ensure a successful scale-up. Future studies will focus on scale-up strategies, formulation and stability studies, and potential applications in functional foods and nutraceutical formulations.

## CRediT authorship contribution statement

**Simona Serio:** Writing – original draft, Validation, Methodology, Investigation, Conceptualization. **Valentina Santoro:** Writing – original draft, Visualization, Validation, Methodology, Investigation, Conceptualization. **Anna Lisa Piccinelli:** Writing – review & editing, Writing – original draft, Visualization, Supervision, Project administration, Methodology, Conceptualization. **Rita Celano:** Validation, Methodology, Investigation. **Luca Rastrelli:** Writing – review & editing, Software, Funding acquisition.

## Declaration of competing interest

The authors declare that they have no known competing financial interests or personal relationships that could have appeared to influence the work reported in this paper.

## References

[1] Alexandri M., Kachrimanidou V., Papapostolou H., Papadaki A., Kopsahelis N. (2022). Sustainable Food Systems: the Case of Functional Compounds towards the Development of Clean Label Food Products. Foods.

[2] Granato D., Bursácbursác F.J.Barba D., Kovačevíc K., Lorenzo J.M., Cruz A.G., Putnik P. (2020). Annual Review of Food Science and Technology Functional Foods: Product Development, Technological Trends, Efficacy Testing, and Safety, Annu. Rev. OfFood. Sci. Technol..

[3] Granato D., Zabetakis I., Koidis A. (2023). Sustainability, nutrition, and scientific advances of functional foods under the new EU and global legislation initiatives. J. Funct. Foods.

[4] Zia S., Khan M.R., Shabbir M.A., Aslam Maan A., Khan M.K.I., Nadeem M., Khalil A.A., Din A., Aadil R.M. (2022). An Inclusive Overview of Advanced thermal and Nonthermal Extraction Techniques for Bioactive Compounds in Food and Food-related Matrices. Food Rev. Int..

[5] Chemat F., Abert-Vian M., Fabiano-Tixier A.S., Strube J., Uhlenbrock L., Gunjevic V., Cravotto G. (2019). Green extraction of natural products. Origins, current status, and future challenges, TrAC -. Trends Anal. Chem..

[6] Herrero M. (2024). Towards green extraction of bioactive natural compounds. Anal. Bioanal. Chem..

[7] Chemat F., Vian M.A., Cravotto G. (2012). Green extraction of natural products: Concept and principles. Int. J. Mol. Sci..

[8] Cannavacciuolo C., Pagliari S., Celano R., Campone L., Rastrelli L. (2024). Critical analysis of green extraction techniques used for botanicals: Trends, priorities, and optimization strategies-a review. TrAC - Trends Anal. Chem..

[9] Vinatoru M., Mason T.J., Calinescu I. (2017). Ultrasonically assisted extraction (UAE) and microwave assisted extraction (MAE) of functional compounds from plant materials, TrAC -. Trends Anal. Chem..

[10] Putnik P., Lorenzo J.M., Barba F.J., Roohinejad S., Jambrak A.R., Granato D., Montesano D., Kovačević D.B. (2018). Novel food processing and extraction technologies of high-added value compounds from plant materials. Foods.

[11] Amador-Luna V.M., Montero L., Herrero M. (2023). Compressed fluids for the extraction of bioactive compounds from plants, food by-products, seaweeds and microalgae – an update from 2019 to 2023. TrAC - Trends Anal. Chem..

[12] Plaza M., Marina M.L. (2019). Pressurized hot water extraction of bioactives, TrAC -. Trends Anal. Chem..

[13] Plaza M., Marina M.L. (2023). Pressurized hot water extraction of bioactives. TrAC - Trends Anal. Chem..

[14] López-Salas L., Expósito-Almellón X., Borrás-Linares I., Lozano-Sánchez J., Segura-Carretero A. (2024). Design of experiments for green and GRAS solvent extraction of phenolic compounds from food industry by-products - a systematic review. TrAC - Trends Anal. Chem..

[15] Özkaynak Kanmaz E. (2018). 5-Hydroxymethylfurfural (HMF) formation during subcritical water extraction, Food Sci. Biotechnol.

[16] Kumar K., Srivastav S., Sharanagat V.S. (2021). Ultrasound assisted extraction (UAE) of bioactive compounds from fruit and vegetable processing by-products: a review. Ultrason. Sonochem..

[17] Rao M.V., Sengar A.S., Rawson S.C.K.A. (2021). Ultrasonication - a green technology extraction technique for spices: a review. Trends Food Sci. Technol..

[18] Shen L., Pang S., Zhong M., Sun Y., Qayum A., Liu Y., Rashid A., Xu B., Liang Q., Ma H., Ren X. (2023). A comprehensive review of ultrasonic assisted extraction (UAE) for bioactive components: Principles, advantages, equipment, and combined technologies. Ultrason. Sonochem..

[19] Brassesco M.E., Brandão T.R.S., Silva C.L.M., Pintado M. (2021). Carob bean (Ceratonia siliqua L.): a new perspective for functional food. Trends Food Sci. Technol..

[20] Gioxari A., Amerikanou C., Nestoridi I., Gourgari E., Pratsinis H., Kalogeropoulos N., Andrikopoulos N.K., Kaliora A.C. (2022). Carob: a Sustainable Opportunity for Metabolic Health. Foods.

[21] Serio S., Santoro V., Celano R., Fiore D., Proto M.C., Corbo F., Clodoveo M.L., Tardugno R., Piccinelli A.L., Rastrelli L. (2025). Carob (Ceratonia siliqua) leaves: a comprehensive analysis of bioactive profile and health-promoting potential of an untapped resource. Food Chem..

[22] Weremfo A., Abassah-Oppong S., Adulley F., Dabie K., Seidu-Larry S. (2023). Response surface methodology as a tool to optimize the extraction of bioactive compounds from plant sources. J. Sci. Food Agric..

[23] Uematsu M., Frank E.U. (1980). Static Dielectric constant of Water and Steam. J. Phys. Chem. Ref. Data.

[24] Martín-García B., Pimentel-Moral S., Gómez-Caravaca A.M., Arráez-Román D., Segura-Carretero A. (2020). Box-Behnken experimental design for a green extraction method of phenolic compounds from olive leaves. Ind. Crops Prod..

[25] Erşan S., Güçlü Üstündağ Ö., Carle R., Schweiggert R.M. (2018). Subcritical water extraction of phenolic and antioxidant constituents from pistachio (Pistacia vera L.) hulls. Food Chem..

[26] Pinto D., Vieira E.F., Peixoto A.F., Freire C., Freitas V., Costa P., Delerue-Matos C., Rodrigues F. (2021). Optimizing the extraction of phenolic antioxidants from chestnut shells by subcritical water extraction using response surface methodology. Food Chem..

[27] García-Marino M., Rivas-Gonzalo J.C., Ibáñez E., García-Moreno C. (2006). Recovery of catechins and proanthocyanidins from winery by-products using subcritical water extraction. Anal. Chim. Acta.

[28] Šafranko S., Ćorković I., Jerković I., Jakovljević M., Aladić K., Šubarić D., Jokić S. (2021). Green extraction techniques for obtaining bioactive compounds from mandarin peel (Citrus unshiu var. Kuno): Phytochemical analysis and process optimization. Foods.

[29] Wijngaard H., Brunton N. (2009). The optimization of extraction of antioxidants from apple pomace by pressurized liquids. J. Agric. Food Chem..

[30] Pagano I., Piccinelli A.L., Celano R., Campone L., Gazzerro P., Russo M., Rastrelli L. (2018). Pressurized hot water extraction of bioactive compounds from artichoke by-products. Electrophoresis.

[31] Rivera-Tovar P.R., Torres M.D., Camilo C., Mariotti-Celis M.S., Domínguez H., Pérez-Correa J.R. (2021). Multi-response optimal hot pressurized liquid recovery of extractable polyphenols from leaves of maqui (Aristotelia chilensis [Mol.] Stuntz). Food Chem..

[32] Yamada K., Shimada T., Nishimura M., Hara-Nishimura I. (2005). A VPE family supporting various vacuolar functions in plants. Physiol. Plant..

[33] Ogino H., Ishikawa H. (2001). Enzymes which are stable in the presence of organic solvents. J. Biosci. Bioeng..

[34] Díaz-de-Cerio E., Tylewicz U., Verardo V., Fernández-Gutiérrez A., Segura-Carretero A., Romani S. (2017). Design of Sonotrode Ultrasound-Assisted Extraction of Phenolic Compounds from Psidium guajava L. Leaves. Food Anal. Methods.

[35] Rodrigues S., Fernandes F.A.N., de Brito E.S., Sousa A.D., Narain N. (2015). Ultrasound extraction of phenolics and anthocyanins from jabuticaba peel. Ind. Crops Prod..

[36] Živković J., Šavikin K., Janković T., Ćujić N., Menković N. (2018). Optimization of ultrasound-assisted extraction of polyphenolic compounds from pomegranate peel using response surface methodology. Sep. Purif. Technol..

[37] Hirondart M., Rombaut N., Fabiano-Tixier A.S., Bily A., Chemat F. (2020). Comparison between pressurized liquid extraction and conventional Soxhlet extraction for rosemary antioxidants, yield, composition, and environmental footprint. Foods.

[38] Jacotet-Navarro M., Rombaut N., Deslis S., Fabiano-Tixier A.S., Pierre F.X., Bily A., Chemat F. (2016). Towards a “dry” bio-refinery without solvents or added water using microwaves and ultrasound for total valorization of fruit and vegetable by-products. Green Chem..

[39] Vernès L., Abert-Vian M., El Maâtaoui M., Tao Y., Bornard I., Chemat F. (2019). Application of ultrasound for green extraction of proteins from spirulina. Mechanism, optimization, modeling, and industrial prospects. Ultrason. Sonochem..

