# Supplementary material for: Advanced extraction techniques for sustainable recovery of health-promoting compounds from carob leaves
Source: Ultrason Sonochem. 2025 Dec 6;124:107710. doi: 10.1016/j.ultsonch.2025.107710 (PMC12753491; doi:10.1016/j.ultsonch.2025.107710)
Supplement: Supplementary Data 1 [file mmc1.pdf]

Supplementary Material

of

Advanced extraction techniques for sustainable recovery of health-promoting compounds from Carob leaves

Simona Serio[a,b,1], Valentina Santoro[a,c,1], Anna Lisa Piccinelli[a,c,*], Rita Celano[a,c], and Luca Rastrelli[a,c]

[a] Department of Pharmacy, University of Salerno, 84084 Fisciano, Salerno, Italy

[b] PhD Program in Drug Discovery and Development, University of Salerno, 84084 Fisciano, Salerno, Italy

[c] National Biodiversity Future Center (NBFC), 90133 Palermo, PA, Italy

[1] Co-first authors

**Corresponding author:* Department of Pharmacy, University of Salerno, Via Giovanni Paolo II 132, 84084 Fisciano, Salerno, Italy

Tel.: +39(0)089969794

E-mail address: apiccinelli@unisa.it

Table S1. Box-Behnken design matrix with target response values and predicted and experimental results obtained under optimal PHWE conditions.

run	block	Temperature °C	Static time min	cycle n	EE_Sil mg g^{-1} leaf	EE_TeGG mg g^{-1} leaf	EE_Myr mg g^{-1} leaf	P_Sil g 100 g^{-1} ex	P_TeGG g 100 g^{-1} ex	P_Myr g 100 g^{-1} ex	EE_GA mg g^{-1} leaf	TPC mg GAE g^{-1}	yield g 100 g^{-1}
1	1	100	8	2	11.5	11.3	8.9	4.8	3.9	2.7	4.3	56.9	30.5
2	1	140	5	2	7.7	11.0	9.5	2.6	3.3	2.5	7.3	74.1	33.9
3	1	140	8	3	4.6	8.8	9.0	1.1	2.2	2.2	13.9	113.9	41.3
4	1	60	8	1	7.2	3.6	5.3	4.1	1.8	2.3	2.1	28.2	21.6
5	1	100	11	3	10.0	11.1	9.0	4.3	4.3	2.9	5.0	70.0	30.8
6	1	100	5	3	12.3	12.8	9.6	5.6	4.5	3.0	4.1	66.8	30.4
7	1	60	5	2	9.1	4.0	6.2	4.5	2.0	2.6	2.4	34.2	23.1
8	1	140	11	2	4.6	7.9	9.2	0.8	2.0	2.2	13.6	107.2	38.9
9	1	100	5	1	11.1	7.9	7.3	5.7	3.4	2.7	2.7	38.9	24.7
10	1	100	11	1	9.9	10.4	8.1	4.3	3.8	2.7	3.6	50.5	28.1
11	1	60	8	3	10.4	6.1	8.0	5.1	2.8	3.0	2.9	45.3	25.9
12	1	100	8	2	11.4	12.4	9.3	4.4	4.2	2.8	4.7	65.5	29.4
13	1	60	11	2	10.2	5.4	7.3	5.3	2.5	2.9	2.9	39.0	25.1
14	1	140	8	1	5.3	9.9	8.7	1.6	3.2	2.6	7.8	75.0	33.7
15	1	100	8	2	10.4	10.6	8.5	4.8	4.5	3.0	3.7	63.2	29.9
16	2	100	8	2	11.4	13.3	9.5	4.5	4.5	2.9	4.1	68.6	29.1
17	2	140	5	2	8.4	13.1	10.0	2.5	3.8	2.6	7.3	82.3	35.1
18	2	140	8	3	5.7	11.8	9.5	1.1	2.7	2.1	11.9	109.9	41.7
19	2	60	8	1	8.1	4.0	5.7	3.9	2.0	2.4	2.3	28.1	22.3
20	2	100	11	3	9.6	12.4	9.1	3.5	4.1	2.7	5.2	69.7	30.9
21	2	100	5	3	12.2	13.3	9.3	4.9	4.7	2.9	4.3	66.3	30.6
22	2	60	5	2	9.9	4.8	6.6	4.4	2.1	2.4	2.5	34.7	23.8
23	2	140	11	2	4.7	8.7	8.6	0.9	2.3	2.1	12.3	97.4	38.9
24	2	100	5	1	10.8	8.7	7.4	5.3	3.7	2.8	2.7	45.3	24.7
25	2	100	11	1	8.8	10.0	7.6	3.8	3.9	2.7	3.8	46.4	27.0
26	2	60	8	3	10.7	6.1	7.9	4.8	2.6	2.8	3.1	41.8	26.2
27	2	100	8	2	11.0	12.7	9.1	4.5	4.5	2.9	4.2	62.4	28.8
28	2	60	11	2	10.3	5.8	7.5	4.6	2.5	2.7	3.0	38.6	25.0
29	2	140	8	1	5.9	11.7	8.9	1.8	3.5	2.6	7.8	76.2	32.2
30	2	100	8	2	10.5	12.3	8.8	4.4	4.3	2.8	4.1	63.2	28.4
**Optmimum** (EEs of Sil, TeGG, Mir, GA; P of Sil, TeGG, Myr)					**EE_Sil**	**EE_TeGG**	**EE_Myr**	**P_Sil**	**P_TeGG**	**P_Myr**	**EE_GA**	**TPC**	**yield**
**Predicted values**		**98**	**5**	**3**	11.9	13.2	9.2	5.3	4.4	2.9	3.9	65.3	30.4
95% confidence interval					11.5 - 12.4	11.9 - 14.4	8.9 - 9.5	5.0 - 5.5	4.2 - 4.7	2.8 - 3.0	3.3 - 4.6	61.0 - 69.5	29.4 - 31.3
Desirability			**0.90**		0.96	0.93	0.84	0.92	0.92	0.86	0.84	0.41	0.45
**Experimental value** (mean ± SD, n = 6)					12.2 ± 0.2	13.8 ± 0.4	9.1 ± 0.1	5.2 ± 0.1	4.6 ± 0.3	2.9 ± 0.3	4.1 ± 0.2	60.0 ± 3.0	29.60 ± 0.7
95% confidence interval					12.0 - 12.4	13.8 - 13.9	9.0 - 9.2	5.1 - 5.3	4.4 - 4.8	2.6 - 3.1	3.9 - 4.2	60.3 - 61.4	28.8 - 30.1

Table S2. Box-Behnken design matrix with target response values and predicted and experimental results obtained under optimal probe-UAE conditions.

run	block	% EtOH v/v	Time min	SLR $g\ L^{-1}$	EE_Sil $mg\ g^{-1}$ leaf	EE_TeGG $mg\ g^{-1}$ leaf	EE_Myr $mg\ g^{-1}$ leaf	P_Sil $g\ 100\ g^{-1}$ ex	P_TeGG $g\ 100\ g^{-1}$ ex	P_Myr $g\ 100\ g^{-1}$ ex	EE_diGGs $mg\ g^{-1}$ leaf	TPC $mg\ GAE\ g^{-1}$	yield $g\ 100\ g^{-1}$	EtOH vol L/kg leaf
1	1	50	10	20	12.0	12.8	7.8	4.6	5.2	2.9	3.3	84.1	25.4	25.0
2	1	70	20	60	11.2	10.6	6.7	4.4	4.4	2.6	3.1	78.7	21.2	11.7
3	1	70	15	20	11.5	11.2	6.9	4.1	3.9	2.5	2.8	77.5	23.0	35.0
4	1	30	20	60	2.3	14.1	8.6	0.8	5.0	2.9	10.0	94.8	29.4	5.0
5	1	30	15	100	2.8	12.7	7.9	1.0	4.9	3.0	9.9	90.1	27.8	3.0
6	1	50	15	60	12.3	13.1	8.2	4.3	4.9	2.9	3.7	92.3	25.5	8.3
7	1	70	15	100	10.7	9.9	6.2	4.8	4.6	2.8	2.9	72.0	20.8	7.0
8	1	50	20	100	13.1	13.9	8.6	4.8	5.4	3.1	3.7	95.8	27.2	5.0
9	1	50	15	60	12.3	13.0	8.1	5.1	5.5	3.4	3.5	92.6	26.5	8.3
10	1	50	10	100	11.9	12.1	7.5	5.2	5.6	3.3	3.5	83.1	24.9	5.0
11	1	30	10	60	4.2	12.7	7.9	1.5	5.2	3.0	8.8	87.0	27.2	5.0
12	1	50	15	60	12.2	12.6	7.9	5.1	5.6	3.3	3.5	92.1	26.3	8.3
13	1	50	20	20	12.9	14.3	8.9	4.8	5.4	3.2	3.5	97.8	29.2	25.0
14	1	70	10	60	10.1	9.5	5.9	5.4	5.2	3.1	2.9	72.1	20.2	11.7
15	1	30	15	20	2.7	14.5	9.1	0.9	4.9	3.0	11.0	98.8	30.2	15.0
16	2	50	10	20	12.0	13.6	8.1	4.0	4.9	2.6	3.5	83.1	24.9	25.0
17	2	70	20	60	10.7	10.0	6.4	3.7	4.1	2.3	3.0	78.9	21.7	11.7
18	2	70	15	20	11.9	11.4	6.8	4.8	4.9	2.8	2.5	78.5	23.3	35.0
19	2	30	20	60	1.9	13.8	8.5	0.7	5.1	2.9	9.9	129.1	29.0	5.0
20	2	30	15	100	2.0	12.5	7.8	0.7	4.9	2.9	9.6	89.8	27.5	3.0
21	2	50	15	60	11.7	12.6	7.9	4.7	5.4	3.2	3.7	91.4	26.6	8.3
22	2	70	15	100	10.0	9.4	5.9	5.1	5.1	3.0	2.9	72.9	19.8	7.0
23	2	50	20	100	12.8	13.7	8.6	4.6	5.1	3.1	3.8	87.8	26.2	5.0
24	2	50	15	60	12.0	12.8	8.0	4.7	5.4	3.0	3.7	90.7	25.5	8.3
25	2	50	10	100	11.9	12.7	7.9	4.9	5.6	3.2	3.6	83.5	24.0	5.0
26	2	30	10	60	1.8	12.0	7.7	0.7	4.9	2.7	8.6	84.2	26.1	5.0
27	2	50	15	60	12.2	12.8	8.0	5.0	5.8	3.2	3.7	91.8	25.2	8.3
28	2	50	20	20	12.9	15.3	9.3	4.5	5.4	3.0	3.9	97.8	28.9	25.0
29	2	70	10	60	9.6	8.8	5.3	4.9	4.8	2.7	2.8	67.6	19.6	11.7
30	2	30	15	20	2.5	14.7	9.2	0.9	4.6	2.7	8.8	95.4	28.8	15.0
**Optmimum** (EE Sil, TeGG, MYr, diGGs; P Sil, TeGG, Myr; EtOH vol)					**EE_Sil**	**EE_TeGG**	**EE_Myr**	**P_Sil**	**P_TeGG**	**P_Myr**	**EE_diGGs**	**TPC**	**yield**	
**Predicted values**		**52**	**20**	**100**	12.9	13.4	8.4	4.9	5.4	3.1	3.6	96.7	26.5	
95% confidence interval					12.4 - 13.3	13.1 - 13.8	8.2 - 8.6	4.7 - 5.1	5.2 - 5.5	3.0 - 3.2	3.3 - 3.9	92.0 - 101.4	25.8 - 27.1	
Desirability			**0.83**		0.98	0.71	0.77	0.89	0.78	0.72	0.87	0.47	0.62	
**Experimental value** (mean ± SD, n = 6)					12.5 ± 0.7	13.9 ± 0.6	8.6 ± 0.2	5.2 ± 0.5	5.1 ± 0.5	3.1 ± 0.3	3.9 ± 0.1	97.0 ± 4.9	27.8 ± 0.6	
95% confidence interval					11.7 - 12.9	13.2 - 14.3	8.3 - 8.7	4.9 - 5.5	4.8 - 5.4	2.9 - 3.3	3.8 - 4.0	93.6 - 100.3	27.3 - 28.3	

Table S3. Analysis of Variance (ANOVA) for response variables of PHWE.

	Sil (mg/g leaf)		TeGG (mg/g leaf)		Myr (mg/g leaf)		GA (mg/g leaf)		Sil (% extract)		TeGG (% extract)		Myr (% extract)		Extraction yield		TPC (mgGAE/g)	
	F-value	*p*-value	*F*-value	*p*-value	*F*-value	*p*-value	*F*-value	*p*-value	*F*-value	*p*-value	*F*-value	*p*-value	*F*-value	*p*-value	*F*-value	*p*-value	*F*-value	*p*-value
**A: Temperature**	183.36	0.0002	219.91	0.0001	157.59	0.0002	1794.4	0.0000	1302.55	0.0000	26.72	0.0067	22.69	0.0089	3090.03	0.0000	438.29	0.0000
**B: static time**	39.15	0.0033	1.85	0.2452	0.11	0.7565	126.24	0.0004	141.18	0.0003	5.33	0.0821	1.69	0.2638	99.19	0.0006	0.34	0.0085
**C: cycle number**	15.38	0.0172	31.12	0.0051	67.84	0.0012	150.86	0.0003	0.02	0.8891	8.18	0.0459	3.00	0.1583	554.37	0.0000	2.55	0.0002
**AA**	253.25	0.0001	202.19	0.0001	27.56	0.0063	328.57	0.0001	627.86	0.0000	366.85	0.0000	58.50	0.0016	96.39	0.0006	2.25	0.0478
**AB**	30.04	0.0054	23.32	0.0085	12.08	0.0255	103.33	0.0005	85.41	0.0008	33.12	0.0045	18.37	0.0128	18.38	0.0128	1.21	0.0241
**AC**	19.57	0.0115	7.47	0.0522	14.12	0.0198	72.04	0.0011	42.40	0.0029	27.97	0.0061	37.50	0.0036	46.41	0.0024	5.94	0.0201
**BB**	1.19	0.3373	7.43	0.0526	1.63	0.2702	1.48	0.2905	8.96	0.0402	3.31	0.1432	0.35	0.5879	10.23	0.0329	1.94	0.0526
**BC**	1.26	0.3244	9.60	0.0363	2.86	0.1661	0.04	0.8532	0.18	0.6960	4.74	0.0950	0.37	0.5734	14.65	0.0187	4.59	0.6140
**CC**	11.24	0.0285	4.52	0.1005	8.96	0.0402	0.55	0.4989	1.55	0.2813	1.88	0.2426	0.35	0.5879	5.71	0.0752	1.36	0.1481
**Lack of fit**	0.71	0.7219	1.70	0.3237	0.41	0.9087	5.01	0.0653	3.30	0.1288	1.34	0.4243	0.55	0.8211	3.32	0.1274	15.81	0.5243
**Lack of fit** [a]	0.77	0.6905	2.45	0.1996	0.62	0.7804	4.29	0.0841	3.01	0.1475	1.65	0.3369	0.52	0.8521	3.47	0.1187	1.99	0.3853
**Model**		0.0000		0.0000		0.0000		0.0000		0.0000		0.0000		0.0000		0.0000		0.0047
**R-squared**		97.42		94.57		96.63		97.03		97.67		95.20		92.11		98.65		98.06
**adjusted R-squared**		96.07		91.71		94.85		95.47		96.45		92.67		87.96		97.95		97.03
**R-squared** [a]		96.99		91.15		95.13		96.96		97.60		93.29		91.46		98.51		97.07
**adjusted R-squared** [a]		95.85		88.33		93.27		95.99		96.68		91.16		88.74		97.84		96.14

[a] not significant terms excluded ($p > 0.05$).

Table S4. Regression equations of the fitted models for the response variables, along with the corresponding optimal levels of PHWE factors.

Response variable (Y)	Regression equation [a]	Optimum factors Temperature	Time	N cycle
**Sil (mg/g leaf)**	$-16.6314 + 0.4597\times A + 0.5854\times B + 5.3264\times C - 0.0020\times A^2 - 0.0086\times A\times B - 0.0209\times A\times C - 0.6769\times C^2$	92	5	3
**TeGG (mg/g leaf)**	$-32.3666 + 0.6128\times A + 1.4800\times B + 3.1319\times C - 0.0023\times A^2 - 0.0103\times A\times B - 0.2650\times B\times C$	121	5	3
**Myr (mg/g leaf)**	$-7.3251 + 0.1745\times A + 0.3958\times B + 3.6289\times C - 0.0004\times A^2 - 0.0039\times A\times B - 0.0125\times A\times C - 0.4010\times C^2$	140	5	2
**Sil (g/100 g extract)**	$-4.2450 + 0.2113\times A - 0.0485\times B + 0.9625\times C - 0.0010\times A^2 - 0.0046\times A\times B - 0.0097\times A\times C + 0.0213\times B^2$	82	5	3
**TeGG (g/100 g extract)**	$-11.6474 + 0.2573\times A + 0.3417\times B + 1.2250\times C - 0.0010\times A^2 - 0.0039\times A\times B - 0.0106\times A\times C$	104	5	3
**Myr (g/100 g extract)**	$-1.2604 + 0.0609\times A + 0.1333\times B + 0.6750\times C - 0.0002\times A^2 - 0.0015\times A\times B - 0.0063\times A\times C$	64	11	3
**GA (mg/g leaf)**	$18.6766 - 0.3457\times A - 0.7375\times B - 1.5875\times C + 0.0015\times A^2 + 0.0107\times A\times B + 0.0269\times A\times C$	88	5	1
**TPC (mg GAE/g leaf)**	$40.3557 - 0.464479\times A - 2.52708\times B - 0.86875\times C + 0.00285547\times A^2 + 0.0411458\times A\times B + 0.130625\times A\times C$	140	11	3
**Yield**	$18.4253 - 0.1543\times A + 1.1278\times B + 1.6042\times C + 0.0011\times A^2 + 0.0058\times A\times B + 0.0278\times A\times C - 0.0569\times B^2 - 0.2083\times B\times C$	140	11	3

[a] Only statistically significant effects are considered.

Table S5. Analysis of Variance (ANOVA) for response variables of probe-UAE.

	Sil (mg/g leaf)		TeGG (mg/g leaf)		Myr (mg/g leaf)		diGGs (mg/g leaf)		Sil (% extract)		TeGG (% extract)		Myr (% extract)		Extraction yield		TPC (mgGAE/g)	
	F-value	*p*-value	*F*-value	*p*-value	*F*-value	*p*-value	*F*-value	*p*-value	*F*-value	*p*-value	*F*-value	*p*-value	*F*-value	*p*-value	*F*-value	*p*-value	*F*-value	*p*-value
A: % EtOH	1095.30	0.0000	346.34	0.0000	988.08	0.0000	1164.9	0.0000	449.94	0.0000	3.65	0.1288	2.28	0.2059	625.31	0.0000	48.25	0.0000
B: extraction time	4.86	0.0415	68.72	0.0000	193.61	0.0002	7.13	0.0162	4.61	0.0984	1.72	0.2593	0.11	0.7533	82.62	0.0000	22.20	0.0002
C: solid-liquid ratio (SLR)	2.81	0.1119	59.77	0.0000	111.75	0.0005	0.21	0.6538	3.55	0.1327	2.70	0.1756	4.08	0.1135	47.89	0.0000	2.38	0.1411
AA	975.84	0.0000	126.66	0.0000	326.17	0.0001	326.58	0.0000	230.88	0.0001	27.98	0.0061	18.59	0.0125	45.32	0.0000	4.89	0.0410
AB	8.05	0.0114	0.72	0.4070	1.35	0.3098	3.82	0.0673	1.97	0.2335	3.28	0.1442	3.97	0.1171	1.72	0.2072	4.00	0.0618
AC	2.85	0.1096	0.49	0.4933	4.81	0.0934	0.38	0.5443	1.11	0.3523	0.53	0.5062	0.60	0.4811	1.39	0.2545	0.03	0.8563
BB	0.00	0.9503	0.20	0.6640	0.06	0.8183	0.46	0.5089	0.30	0.6125	0.00	0.9904	1.19	0.3359	1.14	0.3014	0.01	0.9105
BC	0.03	0.8596	0.20	0.6583	1.29	0.3195	0.00	0.8806	1.62	0.2723	2.40	0.1961	2.41	0.1957	4.23	0.0553	0.43	0.5213
CC	2.50	0.1326	26.64	0.0001	48.11	0.0023	0.24	0.6317	0.05	0.8287	0.81	0.4192	0.09	0.7816	9.31	0.0072	1.15	0.2977
Lack of fit	2.48	0.0963	2.46	0.0978	3.47	0.1189	1.39	0.2793	0.88	0.6211	0.84	0.6434	0.62	0.7795	2.02	0.1498	0.77	0.5254
Lack of fit [a]	1.60	0.2107	1.29	0.3150	3.14	0.1383	2.08	0.1083	0.32	0.7291	0.63	0.5407	0.22	0.8008	2.08	0.1037	1.50	0.2314
Model		0.0000		0.0000		0.0000		0.0000		0.0000		0.0001		0.0002		0.0000		0.0000
R-squared		98.86		96.33		96.81		98.62		97.58		71.93		72.90		97.27		81.12
adjusted R-squared		98.34		94.68		95.12		97.99		96.31		59.29		58.64		96.04		72.63
R-squared [a]		98.47		96.09		96.38		98.28		95.74		52.67		47.52		96.27		73.31
adjusted R-squared [a]		98.23		95.27		95.43		98.10		95.25		47.21		41.47		95.49		70.23

[a] not significant terms excluded ($p > 0.05$).

Table S6. Regression equations of the fitted models for the response variables, along with the corresponding optimal levels of probe-UAE factors.

Response variable (Y)	Regression equation [a]	Optimum factors		
		% EtOH	Time	SLR
**Sil (mg/g leaf)**	$-30.4756 + 1.5485\times A - 0.1926\times B - 0.01142\times A^2 + 0.0049A\times B$	58	20	95
**TeGG (mg/g leaf)**	$8.1178 + 0.2848\times A + 0.1465\times B - 0.0671\times C - 0.0037\times A^2 + 0.0004\times C^2$	39	20	20
**Myr (mg/g leaf)**	$4.9794 + 0.1696\times A + 0.0931\times B - 0.0345\times C - 0.0022\times A^2 + 0.0002\times C^2$	38	20	20
**Sil (g/100 g extract)**	$-12.2283 + 0.5852\times A - 0.0049\times A^2$	60	11	24
**TeGG (g/100 g extract)**	$2.0092 + 0.1417\times A - 0.0015\times A^2$	48	16	63
**Myr (g/100 g extract)**	$1.3580 + 0.0739\times A - 0.0008\times A^2$	48	15	63
**diGGs (mg/g leaf)**	$27.6095 - 0.8242\times A + 0.0526\times B + 0.0066\times A^2$	63	10	100
**TPC (mg GAE/g leaf)**	$65.1469 + 0.6696\times A + 1.4500\times B - 0.0120\times A^2$	30	20	60
**Yield**	$25.0486 + 0.1686\times A + 0.2561\times B - 0.0731\times C - 0.0034\times A^2 + 0.0004\times C^2$	33	20	20

[a] Only statistically significant effects are considered.

Fig. S1. Pareto charts of estimated standardized effects of independent PHWE factors on extraction efficiency (EE) of Sil, TeGG, Myr and GA, purity (P) of Sil, TeGG and Myr, extraction yield, and TPC based on Box–Behnken design.[a]

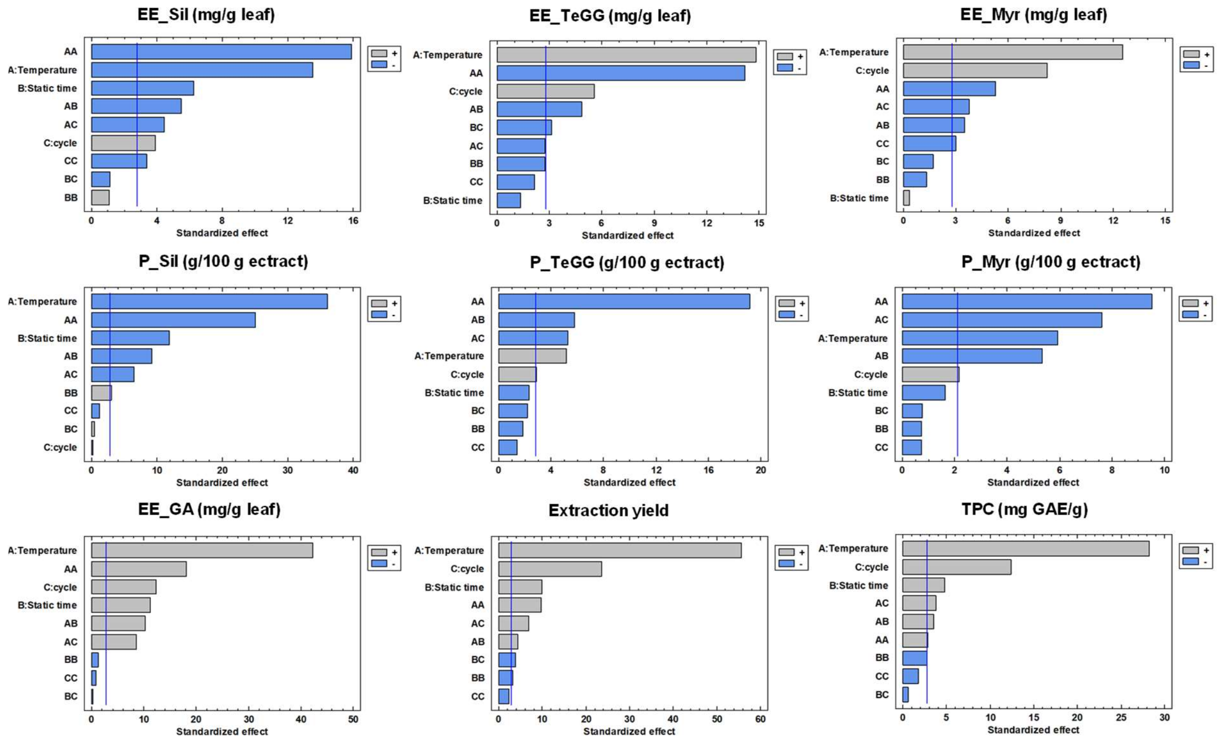

[a] The difference in the bar shadings indicate positive and negative effects of the experimental factors on the response variables, and the vertical line corresponds to the statistically significant with the 95% confidence level.

Fig. S2. Response surface plots of extraction efficiency of Sil (A), TeGG (B), Myr (C) and GA (G); extract content of Sil (D), TeGG (E) and Myr (F); extraction yield (H); and TPC (I) as a function of temperature and cycle for PHWE of bioactive compounds from *Ceratonia siliqua* leaves.[a]

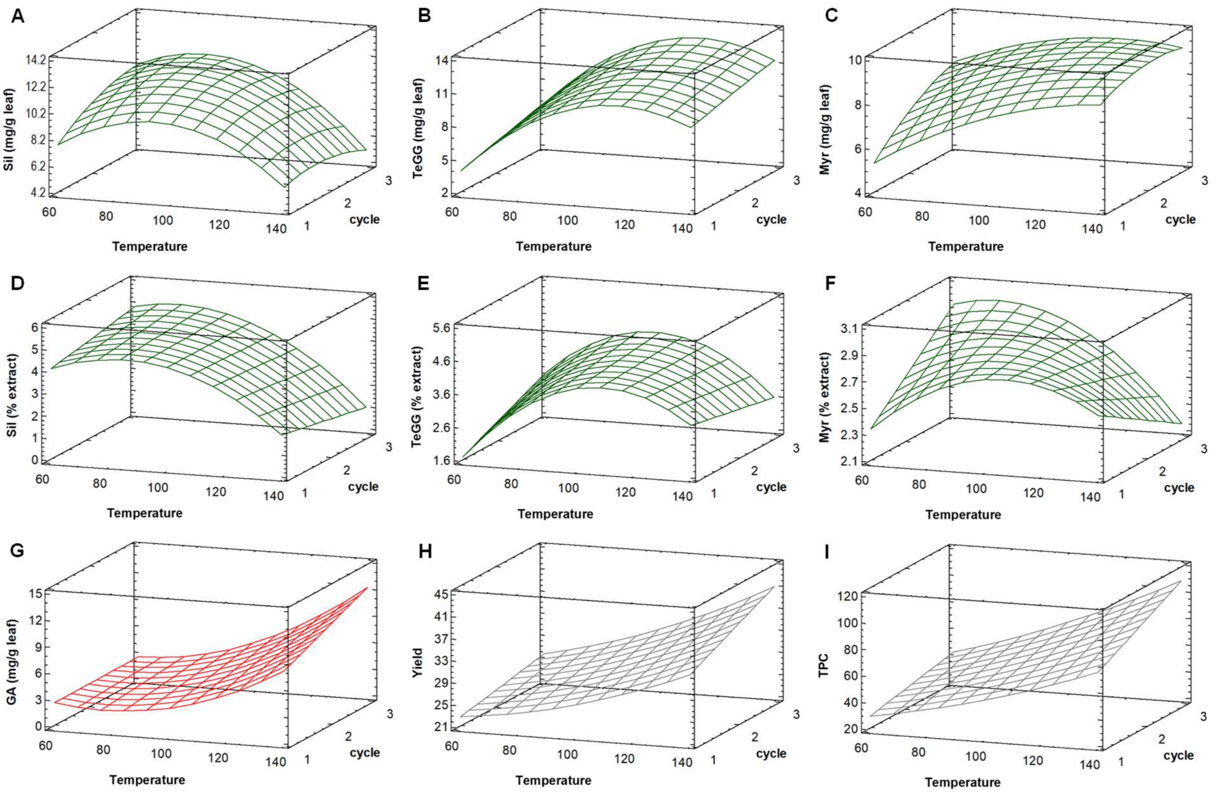

[a] The static time was kept constant at the center point (8 min). Only statistically significant effects are considered.

Fig. S3. Desirability plots of PHWE optimization.

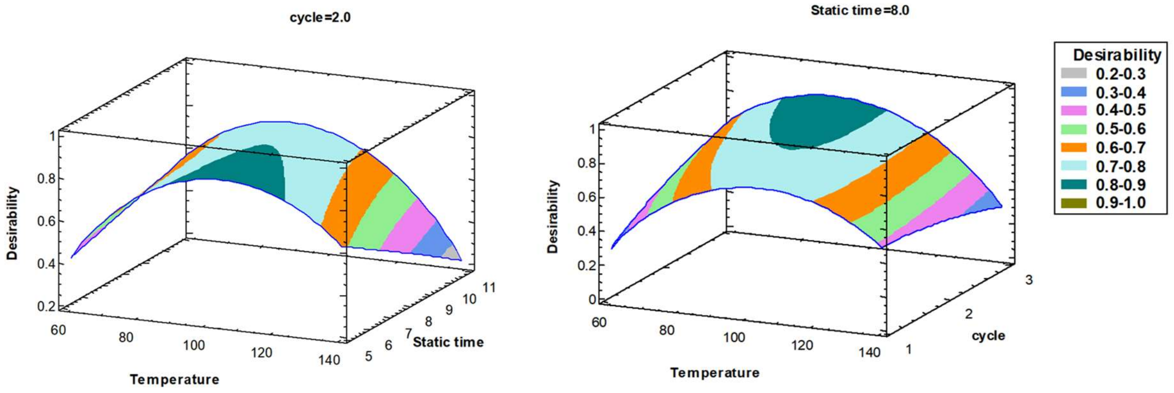

Fig. S4. Pareto charts od estimated standardized effects of independent UAE factors on extraction efficiency (EE) of Sil, TeGG, Myr and GA, purity (P) of Sil, TeGG and Myr, extraction yield, and TPC based on Box–Behnken design.[a]

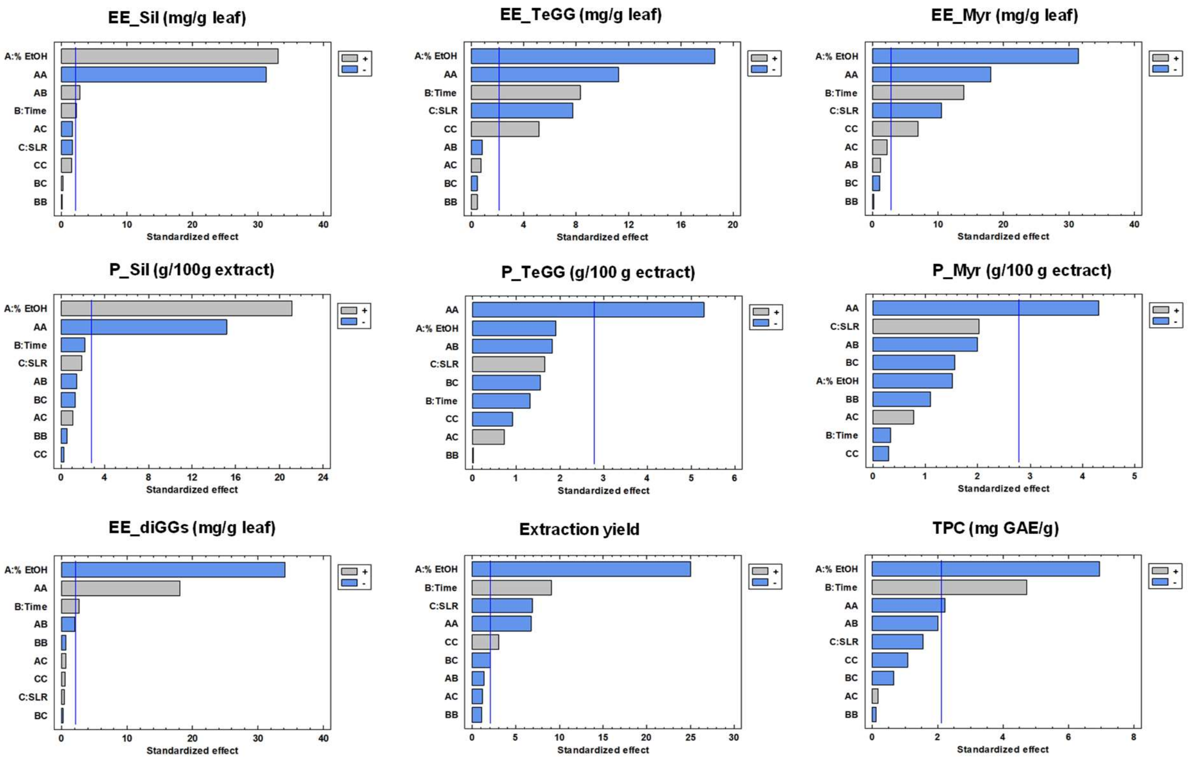

[a] The difference in the bar shadings indicate positive and negative effects of the experimental factors on the response variables, and the vertical line corresponds to the statistically significant with the 95% confidence level.

Fig. S5. Response surface plots of extraction efficiency of Sil (A), TeGG (B), Myr (C) and GA (G); extract content of Sil (D), TeGG (E) and Myr (F); extraction yield (H); and TPC (I) as a function of % EtOH and solid-liquid ratio (SLR) for probe-UAE of bioactive compounds from *Ceratonia siliqua* leaves.[a]

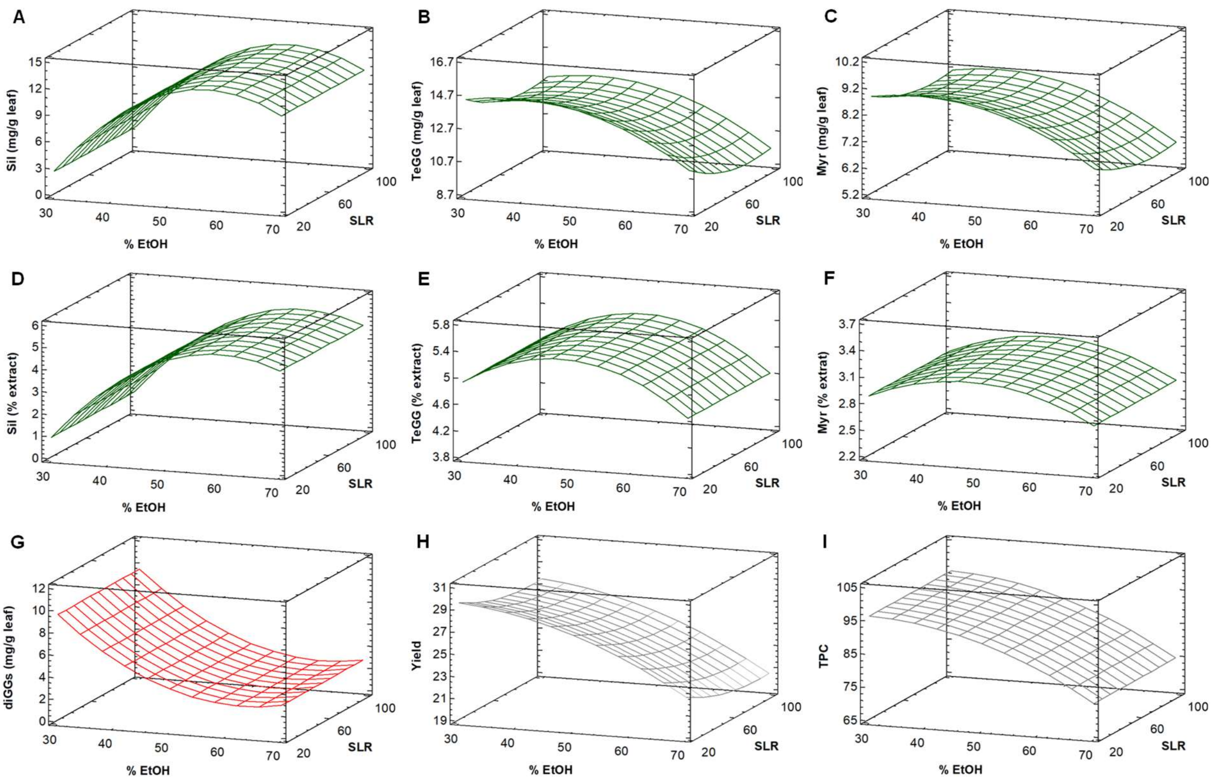

[a] The extraction time was kept constant at the center point (15 min). Only statistically significant effects are considered.

Fig. S6. Desirability plots of UAE optimization.

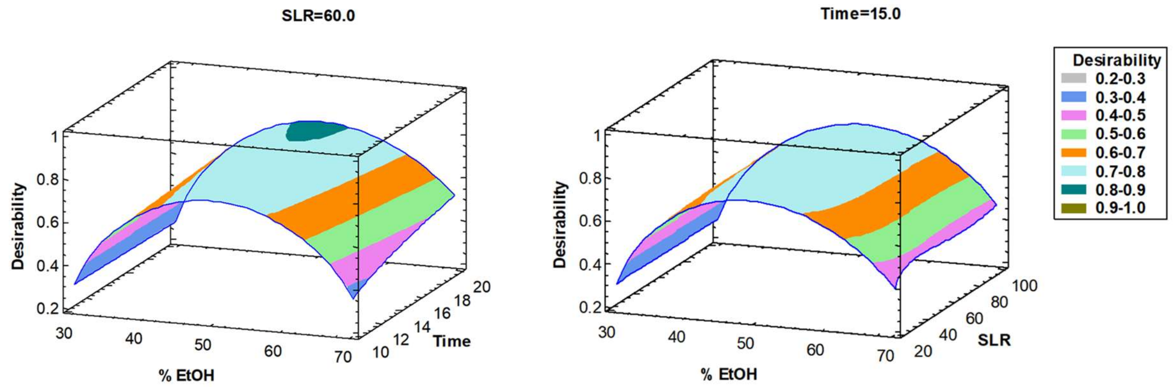

Fig. S7. Effect of PHWE and probe-UAE extracts on cell viability (MTT assay) in HepG2 cell lines, treated for 24h.[a]

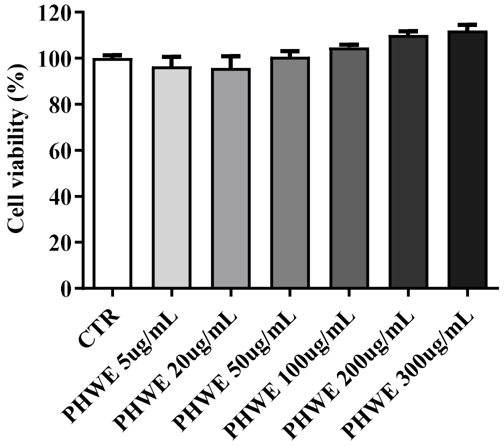

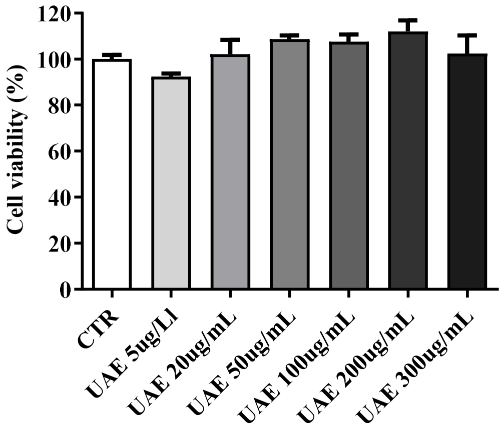

[a] Data are expressed as mean ± SD of at least three independent experiments. *$p < 0.05$ vs. control.
